# Microbial Characterization of a Zambian Honey Vinegar

**DOI:** 10.1002/fsn3.4549

**Published:** 2025-01-19

**Authors:** Garth Anton Cambray, Jarmo‐Charles Julian Kalinski

**Affiliations:** ^1^ Rhodes University Grahamstown South Africa

**Keywords:** acetic acid bacteria, cider vinegar, gut microbiome, honey vinegar, natural vinegar, probiotic

## Abstract

Forest Fruits Organic Honey Vinegar (FFOHV) is a spontaneously fermented (yeast) and acetified (Acetic Acid Bacteria—AAB) Miombo Woodland honey vinegar developed in Zambia. Live vinegars containing live microbial cultures are marketed for their probiotic health benefits. The correlation between a well‐developed gut microbiome and human health is well studied and fermented products such as live vinegar containing AAB contribute to a healthy gut microbiome. This study details a metagenomic analysis of stable, bottled FFOHV (Zambia) alongside two commercially available live vinegar products: Bragg Organic Apple Cider Vinegar (BOACV) and Nature's Source Apple Cider Vinegar (NSACV). FFOHV contained representatives of five bacterial and nine fungal genera, compared to BOACV with two bacterial and five fungal, and NSACV containing no bacterial and six fungal genera. FFOHV and BOACV showed a dominance of *Komagataeibacter* bacterial species. The dominant yeast was *Vanrija humicola* present in all three vinegar samples. FFOHV contained greater diversity of genera, with the notable species *Monascus purpureus*—a microbe that produces several health‐enhancing compounds. The analysis showed that FFOHV is a microbially diverse product containing several potentially health‐enhancing microbes. Graphical Abstract Text: This study presents a metagenomic analysis of Forest Fruits Organic Honey Vinegar (FFOHV) from Zambia, compared with two commercial live cider vinegars: Bragg Organic Apple Cider Vinegar (BOACV) and Nature's Source Apple Cider Vinegar (NSACV). FFOHV exhibited a richer microbial diversity, containing five bacterial and nine fungal genera, including the health‐promoting species *Monascus purpureus*. Both FFOHV and BOACV were dominated by *Komagataeibacter* species, with *Vanrija humicola* as the prevalent yeast across all samples. This confirmed FFOHV's unique potential probiotic benefits.

## Graphical abstract

This study presents a metagenomic analysis of Forest Fruits Organic Honey Vinegar (FFOHV) from Zambia, compared to two commercial live Cider vinegars: Bragg Organic Apple Cider Vinegar (BOACV) and Nature's Source Apple Cider Vinegar (NSACV). FFOHV exhibited a richer microbial diversity, containing five bacterial and nine fungal genera, including the health‐promoting species *Monascus purpureus*. Both FFOHV and BOACV were dominated by *Komagataeibacter* species, with *Vanrija humicola* as the prevalent yeast across all samples. This confirmed FFOHV's unique potential probiotic benefits.

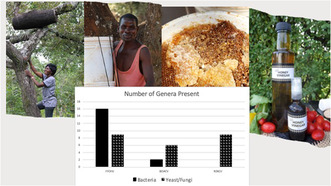

## 1 Introduction

The fermentation of sugar into alcoholic beverages is one of the first bioprocesses developed in most human cultures (Dietler 1990). Mead, the beverage obtained through fermentation of honey (Nigg 1948; Steinkraus and Morse 1966; Crane 1999) is most likely the oldest of these beverages.

Other beverages from ancient times include wine produced through fermentation of fruit (Dupre 1872) and beer through the fermentation of grain sugar obtained via the malting process (Hough, Briggs, and Stevens 1971).

These alcoholic beverages can in turn be converted to vinegar/acetic acid by bacteria from genera such as *Acetobacter*, *Gluconoacetobacter*, and *Gluconobacter* (Chen et al. 2016; Song, Cho, and Baik 2016; Johnston and Gaas 2006; Trček, Mahnič, and Rupnik 2016; Yetiman and Kesmen 2015). In 2012, a significant taxonomic revision took place within the *Gluconoacetobacter* genus, leading to the emergence of a new genus named *Komagatabacter* (Yamada et al. 2012). Consequently, certain species were retained within the original *Gluconoacetobacter* genus, while others found their place in the newly established *Komagatabacter* genus. Over time, the genus has become more commonly known as *Komagataeibacter*, as evidenced by references such as (Yetiman and Kesmen 2015). This taxonomic reorganization reflects the ongoing advancements and refinement in the field of microbiology, providing a clearer understanding of these bacterial groups' relationships and classifications.

The consumption of small quantities of vinegar (a teaspoon) per day is a well‐known traditional remedy in many parts of the world (Budak et al. 2014; Ho et al. 2017). In recent years, “live” vinegar—a term used to describe a vinegar in which a symbiotic culture of bacteria and yeast (SCOBY) and sediments are still present has become more popular due to the purported health benefits of consuming this vinegar ascribed to the beneficial microbes there within. Products such as BOACV (https://www.bragg.com/) have become globally popular due to their emphasis on probiotic microbes. A large number of papers cover the various health benefits of natural vinegars mentioning specifically reducing the impact of likelihood of diabetes, oxidative stress, certain cancers, hypercholesterolemia, as well as boosting the immune system, for example (Johnston and Gaas 2006; Budak et al. 2014; Ho et al. 2017).

Honey represents a diverse source of yeast capable of tolerating high sugar concentrations and fermenting this sugar to alcohol (Cambray 2005). Zambia has a long history of beekeeping which is centered in the biologically diverse miombo forests of Zambia (Clauss 1992). The high temperatures in this region during the flowering season are similar to those cited by (Poot‐Báez et al. 2020) for the Yucatan Peninsula area where Africanized bees are farmed. These authors measured temperatures inside beehives during periods of high ambient temperature and found that hive temperatures could reach as high as 39°C. It is reasonable to conclude that temperatures of both, flowers from which nectar is derived and the beehives in which the honey is produced, will regularly exceed 37°C in the Miombo woodlands.

Forest Fruits Ltd. is a honey processing company in Zambia that sources, extracts, and grades certified organic honey from the Miombo Woodlands of the North‐Western Province through its village beekeeper out‐grower program. It then transports this forest honey to Lusaka, the capital where the honey is graded, packaged, and exported globally.

This honey processing has certain honey grades, and some are too dark in color to market or too strong in taste and consequently excluded from global sales of table honey. High pollen content and dark forest honey are common in this vast biologically diverse forested region and the conversion of these honey products, at temperatures between 28°C and 35°C into organic honey vinegar utilizing a consortium of naturally isolated yeast and bacteria is explored in this article. Typically, vinegar producers use samples of vinegar from other producers to provide starter cultures (Sokollek and Hammes 1997). No existing commercial producers of honey vinegar exist in Zambia, hence, to facilitate the process natural microbes were isolated from honey in conditions where temperatures were between 28°C and 35°C.

It can be surmised that the microbes present in the honey will likely need to be adapted to thrive in these warm temperatures which are in a similar temperature range to the human gastrointestinal tract. This fact informs the choice of fermentation temperatures ideally used to produce honey vinegar using natural microbes present in Miombo woodland honey. In developing a process to utilize natural microbes in the honey, it made sense to conduct such processes in a temperature range like that of the microbial sources to preserve these microbes. It is anticipated that given that this temperature range is higher than the conditions typically present during the ripening time of apples and barley, that this will influence the types of microbes present in the product.

Vinegar has many health‐promoting properties with organoleptic properties conferred by the starting materials (Johnston and Gaas 2006). Developing a process to produce honey vinegar in Zambia allows for the following:

- The organoleptic properties of organic Miombo Woodland honey from Zambia to be incorporated into a vinegar.
- The temperature‐tolerant natural microbes present, and health‐promoting properties of these to be combined into an authentic, organic honey vinegar.

The honey vinegar produced is marketed into global markets, allowing for rural revenue generation in an area with very low income. The largest differentiator between this product and others in the market is the natural origin of fermentative and acetifying microbes and the temperature range to which they are adapted.

Forest Fruits, through their development of honey processing and marketing have enabled and developed the livelihoods of 7000 village beekeepers. The development of a honey vinegar value‐addition step allows the multiplication of income generation, further contributing to economic growth, poverty alleviation, and environmental conservation, while contributing to global health enhancement.

In this regard, the microbiology of the honey vinegar production process, together with the technology developed and the anticipated outcomes, represent an interesting intersection between science, economics, and rural development in one of the least developed regions in the world.

Over a period of 7 years, a proprietary method of producing honey vinegar from raw Miombo woodland honey at large scale was developed. Indigenous consortia of yeast and bacteria were isolated from the beehive components including honey and pollen combs and enriched to enable this process to function. Next‐generation sequencing has been shown to be useful tool to conduct metagenomic analysis of microbial genera, including microbes present in fermented foods and vinegar (Bokulich and Mills 2012; Nie et al. 2013).

Vinegar and honey are widely consumed for their perceived and proven health‐promoting benefits (Ho et al. 2017; Budak et al. 2014; Cianciosi et al. 2018). Honey vinegar combines these 2 traditional remedies into one product.

Literature reports suggest that the benefit of live vinegars is in part due to beneficial organic acids and living probiotics (Budak et al. 2014; Ho et al. 2017). Most of the vinegar globally consumed is produced from temperate crops—crops that are exposed to moderate climatic conditions, and consequently moderate atmospheric temperatures. Crops such as apples (cider vinegar), barley (barley vinegar), grapes (wine vinegar) and rice (rice vinegar) are all crops that grow at an atmospheric temperature at harvest that is below that of the human body.

This article details a preliminary comparative identification of yeast, fungi, and bacterial strains present in marketed Forest Fruits Organic Honey Vinegar (FFOHV) and two commercially available Apple Cider Vinegar products. The two commercially available Apple Cider Vinegar products were selected due to their widespread availability as “standard” commercial bottled live vinegar products.

Due to the above highlighted differences between the feedstocks used to produce the vinegars, it was hypothesized that the honey vinegar microbes would vary considerably in the type and diversity of beneficial microbes to those found in cider vinegar. Given differences in storage conditions, the quantity of detectable microbes present will vary in the samples, hence for a preliminary analysis, the presence or absence of microbes is of most interest, not the quantity.

The objective of this study was to quantify the diversity of microbes present and ascertain if a tropical honey vinegar produced in the 28°C–35°C temperature prevalent in Zambia offers complimentary probiotic health benefits to consumers who consume existing cider vinegars.

## 2 Materials and Methods

For comparative means, two commercially available live vinegar products, Bragg Organic Apple Cider Vinegar (BOACV) and Natures Source Apple Cider Vinegar with Mother (NSACV) were chosen. These products contained a visible SCOBY sediment at the base of the product, with no floating SCOBY present. This SCOBY sediment was sampled.

FFOHV contains two distinct visible layers of microbes, a SCOBY layer that floats and circulates in the bottle, and a sediment that forms at the base. In this regard, a sample of SCOBY and two samples at intermediate levels of sedimentation were sampled. A total of two samples of the commercially available vinegars, and three samples as detailed above of the honey vinegar were taken and subjected to metagenomic analysis.

For bacterial identification, the 16S amplicons were sequenced on a Sequel system by PacBio (www.pacb.com). Raw subreads were processed through the SMRTlink (v11.0) Circular Consensus Sequences (CCS) algorithm to produce highly accurate reads (>QV40). These highly accurate reads were processed through DADA2 (https://benjjneb.github.io/dada2/index.html) and qiime2 (https://docs.qiime2.org/2021.11/) for quality control assessment and taxonomic classification, respectively.

For fungal identification, the ITS1F gene amplicons were sequenced on a Sequel system by PacBio (www.pacb.com). Raw subreads were processed through the SMRTlink (v11.0) Circular Consensus Sequences (CCS) algorithm to produce highly accurate reads (>QV40). These highly accurate reads were then processed through vsearch (https://github.com/torognes/vsearch), and taxonomic information was determined based on QIIME2 results.

The objective of this analysis was to determine the number of Genera and species present, while reliable quantification of these microbes is beyond the scope of this work.

## 3 Results

**FIGURE 1:**
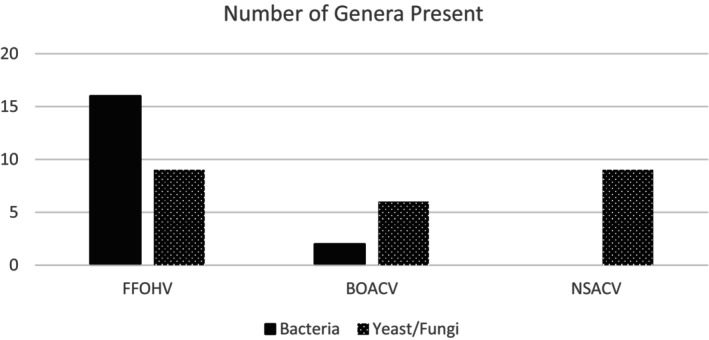
An overview of the genera of bacteria and yeast present in the samples.

To provide a brief overview of the microbial populations identified, it is useful to initially represent the genera present, since many of the microbes were only identifiable to the genus level. It must be noted that actual read counts are not entirely indicative of the actual number of cells because cells may have multiple copies of regions that were amplified. It is however an approximate indication of the presence of cells in specific species or at least genus. The objective of this study was to quantify the species diversity of microbes present, not the quantity of these microbes. The data summarized for genera in Figure 1 indicated a wide diversity of microbes present in FFOHV (16 bacterial and 9 fungal genera), with a slightly narrower diversity present in the BOACV (two bacterial and six fungal genera), and a yet lower diversity within the NSACV which showed a notable absence of bacterial genera (nine fungal genera).

### 3.1 Bacterial Observations

Table 1 shows that for both the FFOHV and BOACV the dominant bacterial genus present was *Komagataeibacter*. The NSACV showed no detectable bacterial presence. The FFOHV showed a significant number of small read count microbes.

**TABLE 1:** Tabulated data for the samples analyzed showing the genera of bacteria detected.

Bacterial genus read counts	FFOHV	BOACV	NSACV
*Komagataeibacter*	27,842	31,258	0
*Sphingomonas*	0	4	0
*Staphylococcos*	4	0	0
*Cutibacterium*	3	0	0
*Bacillus*	58	0	0
*Sporolactobacillus*	15	0	0
*Paenibacillus*	12	4	0
*Brevibacillus*	3	0	0
*Unknown*	16	0	0
*Providencia*	1	0	0
*Rummeliibacillus*	1	0	0
*Enterococcus*	1	0	0
*Uncultured*	1	0	0
*Nodosilinea*	1	0	0
*Nodularia*	1	0	0
*Prevotella*	1	0	0
*Xenococcus*	1	0	0
Genera present	16	2	0

### 3.2 Yeast/Fungal Observations

The data mainly narrow results down to a genus level. This was because a significant number of reads were essentially of unknown species with no sequences lodged.

It must be noted in Table 2 that a rather significant signal was present in all samples for an unknown genus of yeast or fungus. The largest read count of this completely unknown genera and species was present in the Forest Fruits Organic Honey vinegar, with a far smaller number reads of the unknown species present in the BOACV and a few were present in the NSACV. This is an interesting result to investigate further in future research.

**TABLE 2:** Tabulated data analyzed showing the genera of yeast/fungi detected.

Fungal/yeast genus read counts	FFOHV	BOACV	NSACV
*Vanrija*	15,278	8486	4943
*Alternaria*	17	1848	0
*Rasamsonia*	0	473	0
*Unknown*	21,312	465	147
*Unidentified*	2	4	1
*Aspergillus*	34,122	2	3
*Monascus*	28,541	0	1
*Wallemia*	911	0	0
*Cladosporium*	143	0	0
*Mycosphaerella*	870	0	0
*Stemphylium*	0	0	1843
*Penicillium*	0	0	2
*Papiliotrema*	0	0	1
*Neocosmospora*	0	0	1
Genera present	9	6	9

An anomaly in the NSACV was a significant representation by *Stemphylium* genus microbes.

It is also noticeable that the FFOHV showed a very strong signal for the genus *Monascus*, which was further identified to the species level as *Monascus purpureus*. A very weak signal for the same microbe was detected in the NSACV.

A strong signal was detected for *Aspergillus* in the FFOHV with most of these reads being for *Aspergillus heterocaryoticus*. A far weaker signal for the same organism was detected in the BOACV, which also showed a unique, relatively strong signal for *Rasamsonia brevistipitata*.

**FIGURE 2:**
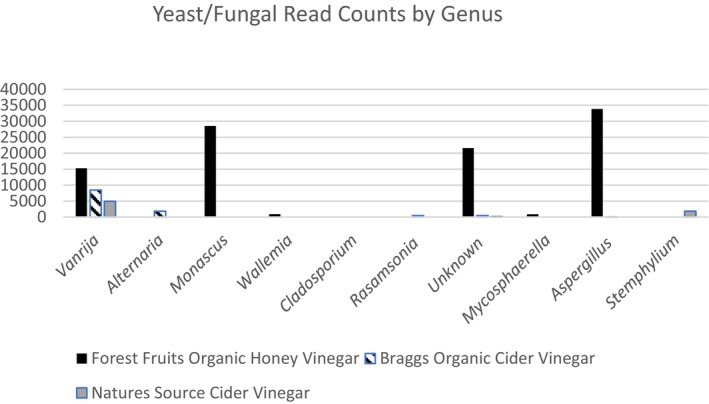
Read counts for the 10 yeast/fungal genera with the highest read counts.

In Figure 2, it is evident that all three samples showed a relatively strong signal for *Vanrija humicola* with wide variation for all other genera present.

## 4 Discussion

Our results indicate a greater diversity of genera of microbes present in FFOHV compared with the two commercial reference products. Of particular interest is the relative increase of certain species, such as *Monascus purpureus* in FFOHV.

It is important to state at this point that this was a broad survey to get an overview of the microbial biodiversity of the FFOHV product and compare this to two commercially available apple cider vinegar products. It is likely that with a larger sample size of each product, that it would be possible to determine relative abundance of microbes. This study did not look at abundance of microbes in any detail, but was mainly focused on the number of genera, and if possible, species of microbe present. Specifically, it aimed to ascertain the presence of potentially beneficial microbes.

In terms of this discussion, it is useful to divide the analysis of the vinegar into the fungal and yeast component as determined by the ITS1 fungal metagenomics probe, and the 16S RNA metagenomics probe of the bacterial populations present.

### 4.1 Observations on the Fungal and Yeast Populations in the Vinegar Samples

*Monascus purpureus* is a fungus traditionally cultivated on rice in China where it produces red pigments, hence it is common name Red Rice Yeast (Yang and Mousa 2012). The pigments produced by *M. purpureus* include dimerumic acid, γ‐aminobutyric acid, and polyketide monacolins such as Monacolin K—Lovastatin (Yang and Mousa 2012). These secondary metabolites have beneficial effects ranging from managing blood pressure and diabetes, dyslipidemia (Yang and Mousa 2012), to obesity and Alzheimer's disease (Shi and Pan 2011). Furthermore, evidence suggests some of these metabolites have beneficial effects in cancer management (Yang and Mousa 2012).

Future metabolomics experiments will determine the presence of these metabolites in FFOHV. *Monascus purpureus*, or Red Rice Yeast, is typically used to produce rice/bran and rice/bran products such as rice vinegar (Ai et al. 2019), that contain the *Monascus purpureus* secondary metabolites. A honey vinegar containing *Monascus purpureus* secondary metabolites will be a unique nongrain‐based source of these metabolites.

*Monascus purpureus* has been shown to be able to lower cholesterol levels by the action of the microbe's metabolism on this chemical in food (Nguyen et al. 2020). The gut microbiome is increasingly being investigated at the fungal/yeast level as the mycobiome, and in this regard shows a high level of variability containing a panoply of fungal, and particularly yeast species, the predominant being *Saccharomyces*, *Malassezia*, and *Candida* species (Nash et al. 2017). The FFOHV represents a relatively novel source of microbial inoculation of *Monascus purpureus* for the mycobiome.

It has been shown that among other fungi, representatives of the genera *Aspergillus*, *Alternaria*, and *Cladosporium* were present in the human mycobiome (Nash et al. 2017)—these genera were also represented to a certain degree in the vinegar samples, with *Aspergillus* and *Cladosporium* represented in FFOHV and *Alternaria* being present in the BOACV.

The presence of *Alternaria angustiovoidea* in the BOACV is most likely due to this being a spore‐forming postharvest spoilage organism in organic apples (Woudenberg et al. 2015). Some *Alternaria* species appear to have a sensitizing effect toward other allergens (Hernandez‐Ramirez et al. 2021). In terms of a live vinegar, this may suggest it to most likely not be a beneficial probiotic, but potentially a problematic contaminant. Some *Alternaria* species, such as *Alternaria alternata* (Hernandez‐Ramirez et al. 2021) are highly allergenic, so the absence of this genus in the FFOHV is not necessarily disadvantageous.

*Vanrija humicola* was present in all three vinegars, with the highest read counts in the FFOHV. This yeast was originally known as *Cryptococcus humicola* but has recently been reclassified (Imanishi et al. 2018). It has been demonstrated that *Vanrija humicola* produces cellobiose lipids that can inhibit the growth of pathogenic *Cryptococcus* and *Candida* yeasts (Kulakovskaya et al. 2009). This is of interest and needs to be investigated further as there appears to be a paucity of information on the presence of such compounds in live vinegars. If such compounds are produced in larger quantities and are present in live vinegar products, the beneficial effects on maintaining healthy gut microbiomes by consuming such vinegar can be ring‐fenced on the molecular level and the exact mechanism of the benefit is then clear to the consumer.

Another interesting, and unique component of the microbiology of the FFOHV, was the presence of *Wallemia canadensis*. *Wallemia canadensis* is regarded as a lower temperature (24°C) *Wallemia* species with slightly fewer extreme requirements than much of the remainder of its genus—this species is not reported to be pathogenic and has been isolated in colder countries from food products in Canada the UK and Finland (Zajc and Gunde‐Cimerman 2018)—which makes its isolation from FFOHV in subtropical Zambia rather anomalous. Some recent reports suggest *Wallemia* species to be present in the gut microbiome of mammals and may indicate good gut health (Li et al. 2020). Currently, there is insufficient data to warrant thorough commentary on the presence of this species in the vinegar other than to note it is not pathogenic and potentially beneficial.

The FFOHV showed a strong signal for *Aspergillus* genus fungi—primarily in the sludge section at the bottom of the bottle. This was dominated by one species, *Aspergillus heterocaryoticum*. Several of the ca. 100 species within the genus *Aspergillus* are pathogens (Paulussen et al. 2017), with *Aspergillus heterocaryoticum* not being known to be one of these. In fact, there is a paucity of literature reports on this species and many *Aspergillus* species are in fact probiotic, with species such as *Aspergillus oryzae* showing many positive impacts on human health (Konishi et al. 2021).

### 4.2 Observations on the Bacterial Populations in the Vinegar Samples

Surprisingly, bacterial populations were undetected in the NSACV probably due to some method of processing such as filtration and fining having removed these. However, both FFOHV and BOACV showed similar dominance of read counts from the *Komagataeibacter* genus.

For both these samples, the identification of *Komagataeibacter* to species level proved unsuccessful. This is rather unexpected as *Acetobacter* species are typically dominant in vinegar production (Gomes et al. 2018). All vinegar samples had been stored in bottles for an indeterminate period, suggesting that *Acetobacter* species may have perished over time and that the specific *Komagataeibacter* species present had prolonged longevity in the bottled environment. As mentioned, both *Komagataeibacter* species in the two vinegar products were unidentified species. It is entirely possible that the NSACV product was acetified with a commercial strain of *Acetobacter*, and that the absence of any detectable bacteria in the NSACV is merely due to the *Acetobacter* species not surviving for long periods of time in bottled live vinegar. It has also been recorded in the natural succession of acetifying bacteria that *Acetobacter* species tend to decline in dominance towards the end of an acetification process and that *Komagataeibacter* species begin to dominate at this point as they are more tolerant of higher acidity (Vegas et al. 2010).

An alternative interpretation may be that microbial guilds (Louca et al. 2018) suited to acetification of alcoholic liquids have demonstrated functional redundancy as has been observed in fermented foods (Leale et al. 2023). In this interpretation of the results, it could be deduced that in the FFOHV and BOACV samples *Komagataeibacter* has performed the functions of early stage acetification that normally would be performed by *Acetobacter* species in conventional systems.

In a comprehensive study, it was noted that one of the dominant microbial genera isolated from Kombucha live SCOBY cultures was *Komagataeibacter* (Harrison and Curtin 2021). These researchers stated that the production of cellulosic materials by the genus is of importance to the SCOBY. They further discussed the impact of “terroir,” or regional geography and ecosystems on the microbiology of fermented products.

Given the large foraging radius represented by the Forest Fruits companies' 7000 beekeepers managing approximately 780,000 beehives over an area of 60,000km^2^, the microbial diversity in this product can be expected to consist of a plethora of species capable of acetifying and fermenting sugars as well as some inactive spores that would enter the process from this large environment.

Bees are known to require a healthy microbiome—the apibiome—to be resistant to diseases (Gorrochategui et al. 2022). The authors found that bees foraging in highly agricultural landscapes, that had suffered extreme flowering species diversity loss, exhibited decreased gut microbiome health compared to bees browsing in natural areas rich in a diverse range of flowering plants. *Komagataeibacter* were found to be an important part of a healthy gut apibiome. The central African forests, including the Miombo woodlands of Zambia, have very high densities of honeybee colonies (Hepburn and Radloff 1998). Commercial agriculture is virtually absent in these areas and most of the land remains natural—a factor that is important in the organic certification of honey products extracted from there. In this regard, we are led to assume that honeybees in these areas would exhibit the healthy gut microbiomes of bees on wild forage as detailed by (Gorrochategui et al. 2022).

Several of the bacteria detected at very low read counts in the FFOHV are worth commenting on. Although present in nearly insignificant numbers it still is interesting to postulate as to the origins of these. *Bacillus circulans* , *Staphylococcus* and *Enterococcus* species, and *Providencia vermicola* were detected in the guts of *Apis mellifera* subspecies sampled in the Kingdom of Saudi Arabia (Khan et al. 2017). The presence of very small read number for *Bacillus circulans* unidentified species of *Staphylococcus* and *Providencia* in FFOHV suggests their origin being from the fact that honey is produced by bees that can harbor such organisms.

Bacteria of the genus *Sphingomonas* and *Prevotella* were found to be present in propolis (Garcia‐Mazcorro, Kawas, and Marroquin‐Cardona 2019)—the FFOHV uses honey gathered by breaking combs harvested from propolis rich bark hives providing a plausible explanation for the presence of a few cells or spores of these microbes in the FFOHV. A species of the genus *Rummeliibacillus* was isolated from spoiled vinegar (Li et al. 2019)—noting that the bacterium was alcohol and salt tolerant. Its presence in FFOHV, given the nature of honey fermentation and acetification, is consequently not surprising.

Various pieces of evidence of honey‐gathering stretching back at least 40,000 years in human history led to Crittenden 2011., hypothesizing that the high sugar, fat, and protein content of beehive contents (honey, brood, pollen) would have enabled the greater brain size of early *Homo* species to be sustained. The evidence supporting a long history of honey gathering, includes analogous behavior by other closely related species such as *Pan troglodytes troglodytes* , as well as other apes (Sanz and Morgan 2009). The authors documented the use of tools by wild chimpanzees engaged in honey gathering. (Crittenden 2011) goes so far as to suggest that in early members of the genus *Homo*, the nutrients (including microbes) present in beehive components may have been critical to the development of a larger brain. It has been observed that a well‐developed and correctly populated gut microbiome correlates to higher cognitive function in 45‐month‐old children (Streit et al. 2021). It is consequently tempting to hypothesize that the acetic acid and other bacteria and fungi in the honeybee microbiome can be a source of beneficial microbes for humans that contribute to overall health.

## 5 Conclusions

In southern African cultures, the symbolism of bees as “the wise ancestors” may shine a light onto the scientific fact that the average human microbiome is increasingly impoverished. Looking to the land and traditions of our origins may be a key to the maintenance of our health and future ability to evolve.

In Zambia the understanding of microbial populations in the traditional fermented milk product, Mabisi, has demonstrated the effectiveness of research in adding value to traditional African fermented products (Moonga et al. 2019). Similarly developing honey products from biologically diverse Zambian forests that allow the export of this apibiome to the global human population may be a tool that allows for the simultaneous development of Africa and the world—providing capital to microbially healthy people in Africa, and microbial health to wealthy, microbially deficient people in the rest of the world.

FFOHV is produced by cultivating the natural yeasts and microbes in honey derived from the Miombo Woodlands of Zambia. As previously mentioned, this region has a mean temperature of 24°C–27°C. Beehives maintain a temperature between 34°C and 38°C (Hammer, Le, and Moran 2021), which is close to the human body temperature range of 36.1°C–37.2°C (Dakappa and Mahabala 2015). Hence, the microbes present in honey are adapted to thrive in temperatures covering the human body temperature range and the diversity of microbes in the FFOHV are consequently valuable to enhance the gut microbiome.

FFOHV is more microbially diverse than the BOACV and NSACV. The presence of several recognized probiotic microbes in FFOHV demonstrates that these microbes can be cultivated from wild‐harvested Zambian honeycomb. These microbes have the potential to contribute to enhancing the gut microbiome of consumers of the vinegar, and in this way improve the overall health of the consumer. In this way, FFOHV represents a complimentary health vinegar that can work together with existing vinegars such as BOACV and NSACV to enable consumers to further enrich their gut microbiomes.

## Author Contributions

**Garth Anton Cambray:** conceptualization (equal), data curation (equal), formal analysis (equal), funding acquisition (equal), investigation (equal), methodology (equal), writing – original draft (lead), writing – review and editing (lead). **Jarmo‐Charles Julian Kalinski:** methodology (equal), writing – review and editing (supporting).

## Ethics Statement

The authors have nothing to report.

## Consent

Written informed consent was obtained from all study participants.

## Conflicts of Interest

The authors declare no conflicts of interest.

## Transparency Statement

Cambray G.A. employed as consultant to study the microbial consortia present. The funders had no role in the design of the study; in the collection, analyses, or interpretation of data; in the writing of the manuscript; or in the decision to publish the results.

## Data Availability

Data are available and uploaded (but embargoed until publication—it can be provided to reviewers).
